# The Irony of Humanization: Alemtuzumab, the First, But One of the Most Immunogenic, Humanized Monoclonal Antibodies

**DOI:** 10.3389/fimmu.2020.00124

**Published:** 2020-02-14

**Authors:** David Baker, Liaqat Ali, Gauri Saxena, Gareth Pryce, Meleri Jones, Klaus Schmierer, Gavin Giovannoni, Sharmilee Gnanapavan, Kathleen C. Munger, Lawrence Samkoff, Andrew Goodman, Angray S. Kang

**Affiliations:** ^1^Blizard Institute, Barts and The London School of Medicine and Dentistry, Queen Mary University of London, London, United Kingdom; ^2^Department of Biological Sciences, National University of Medical Sciences (NUMS), Rawalpindi, Pakistan; ^3^Clinical Board: Medicine (Neuroscience), The Royal London Hospital, Barts Health NHS Trust, London, United Kingdom; ^4^Department of Neurology, University of Rochester Medical Center, School of Medicine and Dentistry, Rochester, NY, United States; ^5^Centre for Oral Immunobiology and Regenerative Medicine, Institute of Dentistry, Barts and The London School of Medicine and Dentistry, Queen Mary University of London, London, United Kingdom

**Keywords:** anti-drug antibodies, CD52, humanized, immunoglobulin, immunogenicity, multiple sclerosis, neutralizing antibodies

## Abstract

Alemtuzumab was designed to reduce the immunogenicity of the parent CD52-specific rat immunoglobulin. Although originally marketed for use in cancer (Mabcampath®), alemtuzumab is currently licensed and formulated for the treatment of relapsing multiple sclerosis (Lemtrada®). Perhaps due to its history as the first humanized antibody, the potential of immunogenicity of the molecule has been considered inconsequential, and anti-drug antibodies (ADA) responses were similarly reported as being clinically insignificant. Nonetheless, despite humanization and depletion of peripheral T and B cells, alemtuzumab probably generates the highest frequency of binding and neutralizing ADA of all humanized antibodies currently in clinical use, and they occur rapidly in a large majority of people with MS (pwMS) on alemtuzumab treatment. These ADA appear to be an inherent issue of the biology of the molecule—and more importantly, the target—such that avoidance of immunogenicity-related effects has been facilitated by the dosing schedule used in clinical practice. At the population level this enables the drug to work in most pwMS, but in some individuals, as we show here, antibody neutralization appears to be sufficiently severe to reduce efficacy and allow disease breakthrough. It is therefore imperative that efficacy of lymphocyte depletion and the anti-drug response is monitored in people requiring additional cycles of treatment, notably following disease breakthrough. This may help inform whether to re-treat or to switch to another disease-modifying treatment.

## Introduction

Alemtuzumab is a monoclonal antibody that is specific for the 21–28 kDa lymphocyte cell surface CD52 glycoprotein (1, 2). This was the first example of a humanized monoclonal antibody (mAb) (3). The initial formulation (Mabcampath® 1,033 mg over 12 weeks) was used to treat CD52+ T and B cell cancers, notably chronic lymphocytic leukemias, and other lymphocyte-mediated conditions (1, 2, 4). However, it is now formulated (Lemtrada® 36–60 mg over 3–5 days over 13 months) and licensed for the treatment of relapsing multiple sclerosis (MS), which is a demyelinating, probable autoimmune disease of the central nervous system (5, 6).

Humanization was a process designed to reduce the immunogenicity of therapeutic monoclonal antibodies that had been generated in rodents (1–3). Although removing rodent constant regions and grafting the complementarity-determining regions onto human framework regions clearly reduced immunogenicity (1), it was soon recognized that alemtuzumab could generate anti-idiotypic responses that could prevent therapeutic benefit (4, 7). Subsequently, perhaps in recognition of the problem of antibody neutralization (8), strategies were developed to limit anti-globulin responses to alemtuzumab (8–10). The occurrence of binding antibodies (BAbs) received limited mention and notably neutralizing antibodies (NAbs) were not discussed in the published reports (5, 6, 11) of the pivotal trials leading to the licensing and commercial development in MS. The first mention of neutralizing antibodies did not occur until we reported on them in 2017 (12, 13). They were described as “inhibitory antibodies” within the regulatory submissions (5, 6). According to the Food and Drug Administration (FDA), their effect on the clinical efficacy and safety profile was of unclear clinical significance. We are concerned that the effect of alemtuzumab anti-drug antibodies (ADA) on efficacy has yet to be adequately addressed (12, 14, 15), and may have safety implications (6, 13, 16, 17). Although ADA against alemtuzumab have been reported as being without clinical significance (14, 15), the dosing at intervals of 12 months or longer may have aided development of alemtuzumab by allowing ADA to subside (Table 1).

**Table 1:** Alemtuzumab dosing schedule can limit anti-drug antibody activity.

**Dosing schedule**	**Observed effects (CARE-MS-Trials)**	**Biology that avoids ADA effects**
First infusion cycle^*^ Five daily 6 h, 12 mg alemtuzumab infusions	ADA develop in most pwMS. (BAbs 62%, NAbs 54% in 1 month)^**^	Primary antibody response usually takes at least 6 days to generate#, **Influence of NAbs avoided**.
Second infusion cycle^*^ Three daily 6 h, 12 mg alemtuzumab infusions	ADA develop in most pwMS. (BAbs 83%, NAbs 79 % in 1 month)^**^	Secondary antibody responses often take 3–4 days to generate#. **Influence of NAbs avoided**.
Repeat infusion cycles ^*^A minimum 12 month intervals *Retreatment after disease activity* **At least 12 months from last dose**	ADA slowly subside with time. Pre-cycle 1. BAbs 0.9% NAbs 0%^**^ Pre-cycle 2. BAbs 29% NAbs 0.6%^**^	Repeat dosing during high titers of BAbs and NAbs avoided. **Influence of ADA avoided**.
*Original schedule 2012–2017* Two cycles^*^	ADA may become more persistentPre-cycle 3. BAbs 75% NAbs 31%^**^(Results at 23 months)	ADA levels wane before next cycle. **Influence of NAbs avoided**.
*New schedule 2017 onwards*, **≤** **four cycles (EU)**, **≤** **three cycles (UK)**		**Pre-dose NAbs may be more problematic for some pwMS**.
Prophylactic anaphylactoid treatment^*^ Anti-histamines, paracetamol, steroids	Reduction of infusion reactions/cytokine release syndrome.	**Masks anaphylactoid responses**, which occur rarely^*^

Dosing schedule of alemtuzumab

* (5), the occurrence of anti-drug antibodies (ADA), binding (BAbs), and neutralizing (NAbs) and adverse effects in people with multiple sclerosis (pwMS) following the treatment cycles in the pivotal CARE-MS I and II trials

** *(12, 13) and the biology, such as the kinetics of antibody formation (18, 19), which could influence the generation and/or action of ADA. European Union (EU), United Kingdom (UK). Pre-cycle refers to the results obtained 1 month before the next infusion cycle, unless otherwise stated as cycle 3 may be ≥24 months (20). Bold letter within the table in column 1 indicates the dosing schedule and in column 3 indicates the influence of NAbs*.

### Alemtuzumab and Anti-drug Antibody Responses

While alemtuzumab (CAMPATH-1H) was originally designed to reduce the immunogenicity of the parent CAMPATH-1G rat immunoglobulin (1–3, 21) with alemtuzumab, ironically this appears not the case, as seen in this comparison among antibody therapies used in MS (Table 2). Moreover, alemtuzumab (36–60 mg Q52W) induces binding ADA in about 85% of cases within 24 months (*n* = 811), and about 92% of those develop neutralizing ADA (12, 13). In the phase III studies, it was evident that, despite substantial lymphocyte depletion, over 60% of pwMS developed ADA within the first month of infusion (12, 13). Furthermore, in the phase II extension study (Maximum *n* = 232), with three cycles of alemtuzumab administered, nearly all of the pwMS eventually developed ADA (8) (Table 1). Even chimeric CD20-depleting antibody (500–1,000 mg rituximab. Q48W) induced ADA in only about 25–37% of pwMS (22, 25). By contrast, humanized ocrelizumab (anti-CD20. 600 mg Q48W) induced ADA in only 0.4% of people with relapsing MS, with <0.1% of people exhibit neutralizing ADA within 2 years of treatment (23). This low level may not simply be due to the humanization process, as this is in part dose-dependent as lower ocrelizumab doses (20 mg) induce ADA in about 20% of pwMS (26). While this dose induced comparable peripheral blood depletion to the 600 mg dose, repopulation was quicker (26), and possibly allows sparing of B cells within lymphoid tissues that can generate the ADA response. Humanized, natalizumab (anti-CD49d-CD29. 300 mg Q4W) induces ADA in about 5–9% of people with MS (24). These are all significantly less than that of alemtuzumab treatment of pwMS (12) (Table 2).

**Table 2:** A high frequency of anti-drug antibodies develops following alemtuzumab treatment in people with MS.

**Antibody**	**Target**	**Dosing**	**Frequency of ADA**	**References**
Rituximab	CD20	1,000 mg Q26W	BAbs 24–37%	(22)
Ocrelizumab	CD20	600 mg Q26W	BAbs 0.4%, NAbs <0.1%	(23)
Natalizumab	CD49d	300 mg Q4W	BAbs 5–9%	(24)
Alemtuzumab	CD52	36–60 mg Q52W	BAbs 85%, NAbs 78%	(12)

*Reported frequency of anti-drug antibody (ADA) responses to various disease-modifying therapies (infusion dose and frequency are shown) during the first 2 years of use in major clinical trials for multiple sclerosis*.

### Biology Supporting the Generation of Anti-drug Antibodies

**Figure 1:**
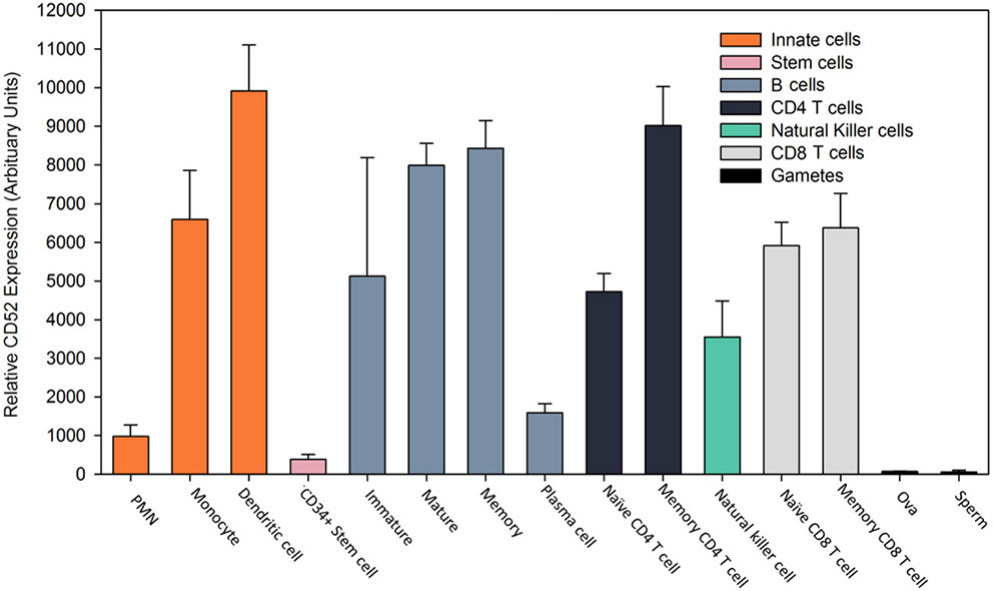
Expression profile of CD52 antigen on leucocytes. Gene expression of CD52 antigen in various cell types assessed using microarray. Data was extracted from the BioGPS portal [www.biogps.org] (30–32) using normalized data from the Primary Cell Atlas (http://biogps.org/dataset/2429/primary-cell-atlas/) (33) and the CD52 probe (34210_at) in Affymetrix Human Genome U133 Plus 2.0 expression arrays (33). The results represent the mean ± SD relative gene expression (arbitrary units) from 2 to 5 replicates. Polymorphonuclear neutrophils (PMN).

The antibody humanization process has been refined since alemtuzumab was first invented, as it may be possible to reduce the immunogenicity of anti-CD52 antibody compared to alemtuzumab (27, 28). However, high frequency of ADA following alemtuzumab infusion may be due to its particular biology, which probably relates to the pattern of CD52 antigen tissue-expression and the depletion/repopulation kinetics of immune cells. Alemtuzumab is (a) given as an effective bolus (5). As CD52 antigen has a wide distribution outside the circulation, the CD52 receptors on leucocytes outside in tissues can absorb the antibody, and this can lead to the relatively short, peripheral blood half-life of alemtuzumab and rapid clearance from the circulation (15). Thus, the cells that escape the initial depletion event are not targeted again until the next cycle of treatment ~12 months later. This is unlike cladribine and ocrelizumab that are administered again 2–4 weeks after the initial dose (23, 29). As such, pwMS who do not deplete lymphocytes effectively after the first dose are more likely to subsequently develop ADA (13, 15); (b) alemtuzumab targets antigen-presenting cells, which include dendritic cells, and B cells, due to their expression of CD52 (Figure 1). Although transiently depleted, monocytes repopulate within a month while circulating antibody is probably still present, and so could rapidly present antigen to remaining antigen-specific lymphocytes, as could any antigen presenting cell that escaped depletion (15, 34). Similar to T cells, surviving B cells (35), could exhibit homeostatic expansion (36), following alemtuzumab-induced depletion and 1 month after treatment memory B cells remain a significant proportion of the B cell pool (37, 38). These cells can be efficient at presenting antigens, notably their specific antigen (39, 40), and could as a result complement the rapid generation of ADA, probably stimulated by professional antigen-presenting cells and supported by the activity of surviving T cells (41); (c) ancillary molecules are needed for the lytic action of alemtuzumab. These include the need for the complement cascade for complement-fixation or cells for antibody-dependent cellular cytotoxicity to co-localize and enter the target tissue (42). This may explain why it appears that alemtuzumab does not purge the lymphoid tissue and bone marrow effectively, as seen in humanized CD52 transgenic mice (43). As such, sequestration of lymphocytes into lymphoid tissue (and possibly the bone marrow) by fingolimod, due to sphingosine-1-phosphate receptor modulation, appears to inhibit the activity of alemtuzumab in some individuals (44); (d) this may allow the B cell niche in the bone marrow to survive and could account for the rapid hyper-repopulation of immature/transitional B cells and naïve/mature B cells that may form the precursors for ADA formation (12); (e) this occurs at a time when CD52 depletion appears to block immune-tolerance induction (12, 45). While it has been reported that the proportions of CD4 T regulatory cells increase compared to CD4 T helper cells (35, 46), in terms of absolute numbers they are dramatically decreased, especially in relation to hyper-populating immature B cells (12, 37). However, CD8 T cells may control this response, and this subset is markedly depleted by alemtuzumab (12, 45). This perhaps creates an environment for ADA generation that occurs with high frequency within the first month of infusion (8, 12, 15). Whether this represents T cell-independent extrafollicular zone directed immune response, as suggested for the formation of ADA to other antibodies (47), is currently unknown. Regulatory cells recover faster than potentially pathogenic memory T and B cells, allowing for control of MS (12). However, this early loss of immune tolerance may also allow the generation of antibody-mediated secondary autoimmunity to develop, which occurs at a high frequency (~40–50%) in pwMS within 5 years from infusion (5, 20, 48). This problem occurs in MS at a higher frequency compared to that observed in cancer following alemtuzumab use (49). Similarly, only 4/211 (1.9%) of people treated for cancer developed ADA (50). This suggests a dose-related difference, or that perhaps the genetics of people with MS and other potential autoimmunities (7) may also predispose them for generating immune responses that may contribute to generating ADA responses; (f) Lastly, since peripheral B cell niches may not be effectively purged, and CD52 is only weakly expressed by plasmablasts and plasma cells (Figure 1) (51, 52), alemtuzumab may not particularly target antibody-forming cells. The low expression of CD52 on plasma cells suggests that once formed, antibody responses (including ADA responses) will persist. Consistent with this view, vaccine responses to common virus and recall antigens persist following alemtuzumab treatment, and the ability to mount responses to novel antigens is retained once the antibody has cleared (53). Thus, ADA titers are boosted with each infusion cycle (15), and this increases the risk of neutralization over time, as the number of treatment cycles increases.

### Anti-drug Antibody Generation May Have Influenced the Treatment Protocol for Alemtuzumab

With the recognition that humanized forms of CAMPATH-1 could still elicit strong ADA responses (4, 7, 54, 55) with a reduction in therapeutic benefit (7, 56), strategies to inhibit ADA to alemtuzumab were investigated (8–10). As such, ADA might have been a consideration in the clinical dosing schedule developed for alemtuzumab in MS (Table 1). Dosing is limited to 5 days for the first treatment cycle and 3 days for the second and subsequent treatment cycles. Repeat dosing has to be 1 year after the last treatment cycle, rather than following disease breakthrough, and only two cycles of dosing were to be initially administered (5). Lastly, infusion of alemtuzumab occurs under anaphylactoid reaction prophylaxis (5, 11, 20).

The dosing schedule may thus avoid potential influences of ADA response. As such, it takes at least 6 days for B cell responses to be generated, with primary antibody levels peaking sometime after that, and it will take a few days to generate an effective secondary antibody response (18, 19, 57). These antibody responses appear to take about a year to subside to near background levels to allow re-dosing (12, 15). Importantly, while there were no pre-treatment NAb responses prior to treatment cycle 1, and only about 0.6% of people had NAbs prior to cycle 2 in the phase III trials, about 31% of people had persistent NAbs, which can limit activity at the end of the second treatment cycle, and over 75% of people had persistent BAbs (12, 13, 15). Anaphylactoid reaction prophylaxis is largely being given to limit the problems of infusion reactions, which are common (>80%), especially during the first cycle of infusions. These are associated with the reactivation of symptoms that occur with pre-existing demyelinated lesions (5, 58). Infusion reactions are largely a product of the cytokine release syndrome, occurring following cell lysis, due to antibody-mediated attacks. The antihistamines and glucocorticosteroids would also mask any potential anti-globulin allergic response, which have not been a significant adverse event (5, 11, 20). While this dosing schedule may have served to avoid the potential issue of antibody immunogenicity, it also generated the concept of pulsed “immune reconstitution therapy” (IRT). This demonstrates that it is possible to get long-term benefit—and possibly long-term remission for some people—from a short treatment cycle, creating a new therapeutic paradigm that did not depend on continuous treatment (48, 59).

### Neutralizing Anti-drug Antibodies Generation May Become More Problematic With Increasing Number of Treatment Cycles and Will Need Monitoring

**Figure 2:**
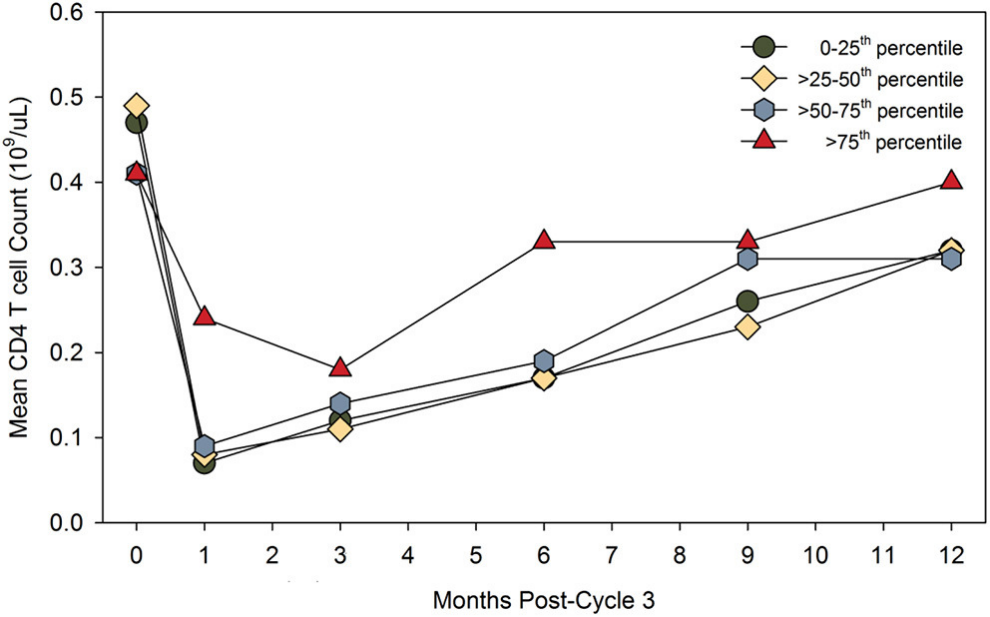
High-titer binding and neutralizing ADA may limit CD4 T cell depletion. People in the CARE-MS extension study received three cycles of alemtuzumab. The results compare the pre-dose binding and neutralization ADA titer, expressed as the lowest to highest quartile and the post-dose absolute number of peripheral CD4 T blood cells over time. The diagram was adapted from data presented in Jacobs et al. (14). The data for the highest quartile was described as “limited and non-significant.” Poster available. https://onlinelibrary.ectrims-congress.eu/ectrims/2018/ectrims-2018/228455/alan.jacobs.minimal.impact.of.anti-alemtuzumab.antibodies.on.the.html (accessed 5th December 2019). Reproduced with permission from L. Chung and Genzyme.

**Figure 3:**
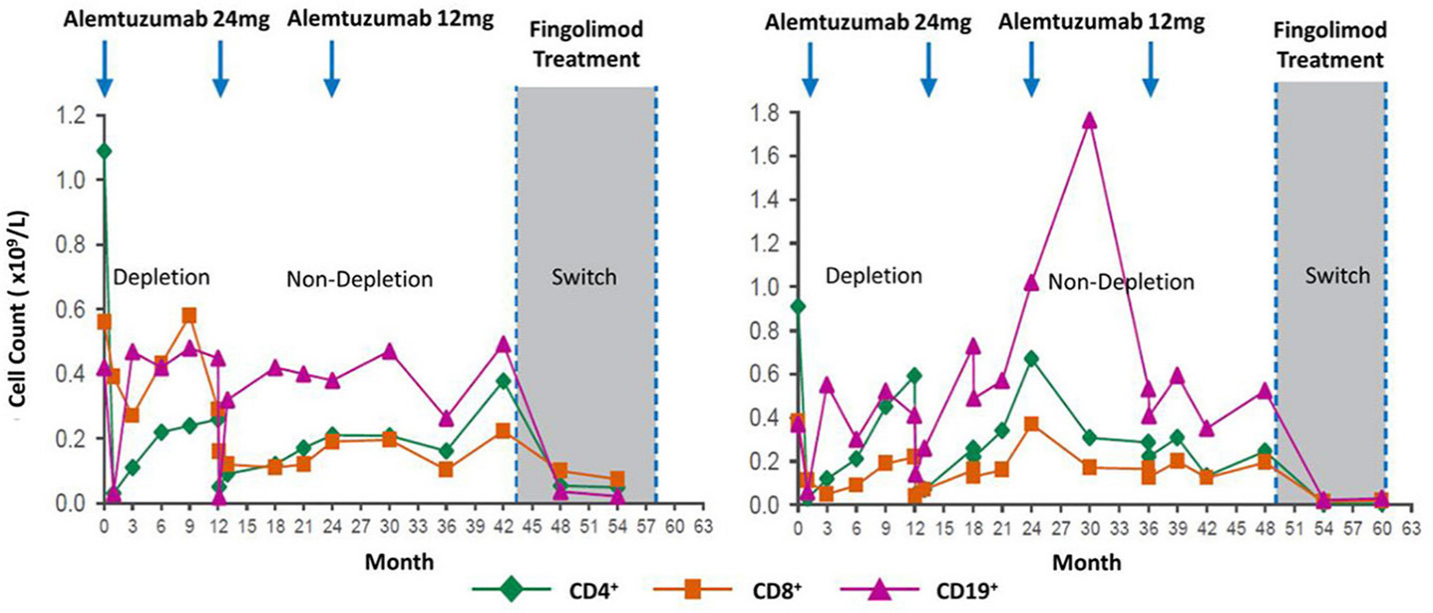
Loss of lymphocyte-depleting function after three alemtuzumab cycles. Lymphocyte depletion following alemtuzumab treatment in people that switched to fingolimod. Evidence for loss of function after two or more cycles. Although the clinical course is not shown, additional treatments after two cycles or switching to another treat in an indicator for disease breakthrough in the form of new relapses or magnetic resonance imaging. Poster available http://www.empireneuro.org/sitebuildercontent/sitebuilderfiles/ean2015poster.pdf (accessed 5th December 2019). Reproduced with permission from Genzyme and D. J. Arnold.

**Figure 4:**
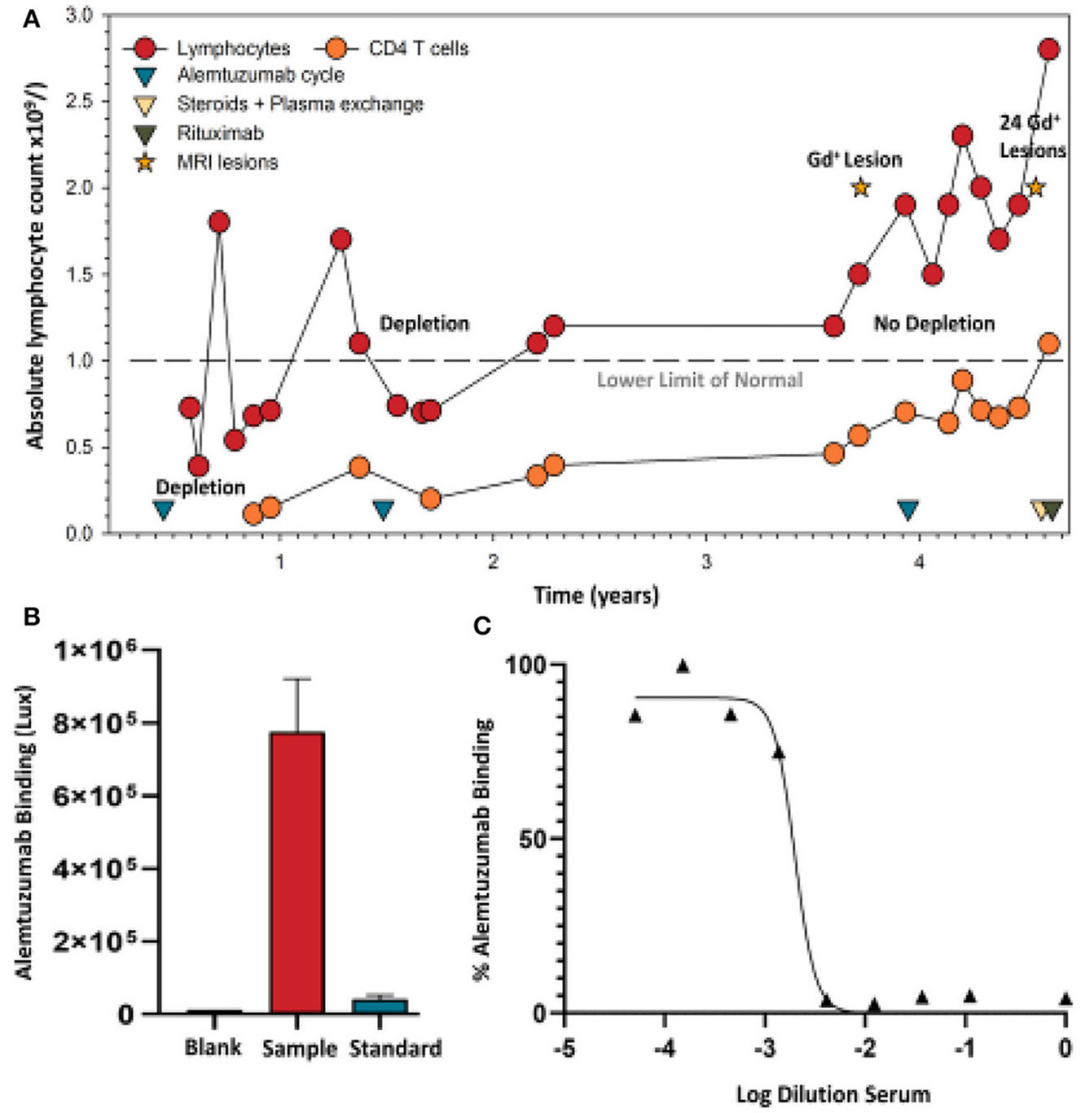
Alemtuzumab neutralizing antibodies develop in a person failing treatment. **(A)** Lymphocyte (lower limit of normal is shown by a dashed line) and CD4 T cell levels were assessed in a person with clinically-definite multiple sclerosis who received the standard 12 mg/day dosing of alemtuzumab at 12-month intervals. Following detection of an active spinal cord lesion, detected by T1 gadolinium (Gd+) enhanced magnetic resonance imaging, an additional alemtuzumab infusion cycle was given. Lymphocyte deletion was limited. A magnetic resonance imaging scan subsequently detected 17 brain and 7 spinal cord gadolinium enhancing lesions, and prompted intravenous methyl prednisolone and plasma exchange, followed by an oral steroid taper. A serum sample (collected during routine sampling) following five cycles of plasma exchange was used following informed written consent and approval given for publication, consistent with institutional guidelines. These were assayed to conform with United Kingdom regulations. **(B)** Binding (Saxena et al., in press) and **(C)** neutralizing ADA (Ali et al., in press) levels taken prior to 1000mg rituximab therapy, which was given at 2-week intervals.

While the frequency of ADA during two cycles is high, the titer generally drops sufficiently to allow effective re-treatment (12, 48). However, with time they may become more persistent (8, 13). As such, ADA could be an issue for any pwMS receiving a third cycle of alemtuzumab, although they have not been a problem at the population level, as alemtuzumab continues to deplete (14, 15, 48, 60). While available data suggests that a lack of response after a third cycle of alemtuzumab is probably only in a minority of pwMS (14), it still appears that those with the highest titer ADA (binding and neutralizing) pre-cycle 3 (>75 percentile) exhibit the poorest lymphocyte depletion potential (Figure 2) (14). People with high-titer neutralizing ADA responses can fail to deplete. This can lead to disease breakthrough and accumulating disability (Figures 3, 4) (8, 13, 16, 17).

Initially, alemtuzumab had a liberal license in Europe, requiring only an active lesion on MRI for use (15). More recently, vascular side effects following infusion has moved alemtuzumab largely to a second-line status in the European Union, and it remains largely a third-line treatment in the United States, where it has remained ever since receiving FDA approval (15, 61). However, as the third and potentially fourth treatment cycle of alemtuzumab was approved by the European Medicines Agency (EMA) in 2017, and the National Health Service (NHS) in the United Kingdom in 2018 (15, 62), measurement of ADA would be more important to help inform re-treatment or drug-switching decisions for individual patients.

If using alemtuzumab, it is imperative that lymphocyte depletion is assessed. This also applies to any other lymphocyte-depleting agent, as people fail to reduce lymphocyte levels in response to treatment with a variety of agents, probably due to their genetics (13, 63). Although it has consistently been reported that total lymphocyte levels do not predict disease activity (15, 60), lack of depletion can be seen to be associated with disease breakthrough and treatment failure in individuals, necessitating a switch to alternative therapies (Figure 3). Such individuals could be found in meeting reports (8, 16, 17). However, the scale of the issue needs to be addressed, although presumably the frequency of total lack of efficacy is low (14, 48). Nonetheless, in one study, two out of six people switching to fingolimod could be seen to totally fail to deplete prior to switching (Figure 3) (16)—this suggested the presence of neutralizing ADA. Appreciation of this issue could possibly spare individuals from unnecessary disease activity and disability that untreated MS causes.

### Alemtuzumab Screening Assays

At present, alemtuzumab ADA assays are neither routinely supplied by the manufacturer nor required by regulatory authorities. However, the reagents with which to construct an assay for alemtuzumab ADA and a neutralizing assay are commercially available. To support clinical use of alemtuzumab in our clinical practice—as it is a valuable treatment for many people with MS, and the possibility of third and fourth courses of treatment were available (5, 48)—we developed a novel assay to detect ADA against alemtuzumab, using a synthetic recombinant construct Alem GloBody (64). This consists of the alemtuzumab variable heavy (V_H_) and light (V_L_) domains held together by an engineered tandem nanoluciferase molecule, such that the V_H_ and V_L_ will pair up and retain antigen binding, and the dual luciferase activity is not compromised (64). In the presence of ADA, the Alem GloBody-IgG complexes form. Since the Alem GloBody lacks the IgG constant domains, it cannot bind to Protein G. However, the complexes can be captured via the Fc of the ADA, and the retained luciferase activity is proportional to the level of ADA in the sample. The assay is performed in <3 h and currently only requires 20 μL of serum. Secondly, we have developed a stable adherent CHO cell line expressing human CD52 for use in a competition assay with sera and alemtuzumab conjugated with Alexa-488 (65). In the absence of neutralizing antibody, the alemtuzumab-Alexa-488 binds to the cells with maximum fluorescence. If neutralizing antibodies are present, they inhibit alemtuzumab-Alexa-488 binding to the cells and the signal is reduced. This reduction in signal can be titrated, and a value assigned to the dilution, requiring giving 50% inhibition (ID_50_). This assay currently requires only 10 μL of serum and takes 3 h (65). These two assays can be used to detect the development of binding and neutralizing alemtuzumab-specific ADA, as seen in an individual with MS (Figure 4) who stopped depleting lymphocytes and exhibited breakthrough disease activity as indicated by new lesion formation (Figure 4A). In comparison to untreated serum (baseline 1.22 x 10^4^ Lux), blank, and a 50 μg/mL anti-alemtuzumab standard (4.66 × 10^4^ Lux), the serum had a very high titer (>7.7 × 10^5^ Lux, despite five cycles of plasma exchange) of binding (Figure 4B), and neutralization (Figure 4C) of ADA could be detected. Although this does not prove cause and effect, it is inconceivable that high titers of neutralizing antibodies are without any influence if there are pre-existing ADA. It has been suggested that ADA are without significant influence (5, 11, 14). While this may be the case at the population level (14, 60), this seems not the case for certain individuals (13). This study indicates that neutralization of alemtuzumab occurs and appears to be clinically relevant in some individuals. Thus, the monitoring of ADA responses may be helpful in the decision to re-treat or switch treatments.

## Conclusions

High-titer neutralizing ADA responses can be associated with a lack of clinical response (8, 13, 17) (Figure 4). These can become high-titer and persist for years (unpublished observations). However, it remains to be determined whether there is a pre-dose antibody-titer limit, above which further dosing is unlikely to work effectively. People within the phase III CARE-MS trial (*n* = 712), and extension studies (*n* = 292), had their ADA (binding and neutralizing) and lymphocyte levels monitored (14)—suggesting that the manufacturer could address this point. Based on our findings, it would seem important for 1-month pre-dose neutralizing ADA titers relating to post-dose lymphocyte depletion failure to be reported, in order to evaluate the concerns raised here. Further, ongoing studies on assay development and validation are in progress and are required to define prognostic ADA levels that may predict lymphocyte depletion and potential treatment failure, such that they can inform on re-treat or switching options. We believe pre-dose screening should be offered and adopted, and that the switching of treatments should be instigated where relevant, as it is important that further neurological disability is not accumulated because patients are being given an ineffective treatment. Here, we demonstrated the utility of GloBody™ for alemtuzumab ADA detection. GloBody reagents based on other antibody-therapeutic binding sites may be generated (including for CART-cell), and the generic platform may be adopted to monitor ADA responses (64).

## Data Availability Statement

The datasets generated for this study are available on request to the corresponding author.

## Ethics Statement

Ethical review and approval was not required for the study on human participants in accordance with the local legislation and institutional requirements. The patients/participants provided their written informed consent to participate in this study.

## Author Contributions

AK and DB conceived the concept and drafted the initial manuscript. AK designed the GloBody molecule and assays. GS, LA, MJ, and GP developed the cell line, prepared the reagents, and developed and applied the assays. KM, LS, AG, SG, KS, and GG provided the clinical samples. All authors discussed the results and contributed to the final manuscript.

### Conflict of Interest

AK has trademarked GloBody™ and filed patents for potential commercial development related to the Globody™ ADA technology. AG, GG, SG, and KS have received fees for consultancy, meetings, and grant support (SG) from Sanofi Genzyme within the last 3 years, otherwise none are considered relevant. However, SG has received travel support, consultancy fees, or grant support from Biogen, Novartis, Teva, Pfizer, and Takeda. DB has received consultancy and presentation fees from Canbex Therapeutics, Japan Tobacco, Merck, and Roche. KS has received consultancy and presentation fees from Biogen, Bayer HealthCare, Lipomed, Medday, Merck, Novartis, Roche, and Teva. GG has received consultancy, presentation fees, or grants from Abbvie Biotherapeutics, Actelion, Atara Bio, Biogen, Canbex, Celgene, Genentech, Japan Tobacco, Merck, Novartis, Roche, Sanofi-Genzyme, Synthon, Takeda, and Teva. AG has received fees for consultancy from Abbvie, Acorda Therapeutics, Adamas, Atara, Bayer, Biogen Idec, Celgene, Genentech, Sanofi Genzyme, Mylan, Novartis, and Teva. The University of Rochester (the employer of KM, LS, and AG) has received support for conducting clinical trials from Acorda Therapeutics, Avanir, Biogen-Idec, EMD-Serono, Genzyme, Novartis, Ono, Roche, Sun Pharma, Teva, and Vaccinex. The remaining authors declare that the research was conducted in the absence of any commercial or financial relationships that could be construed as a potential conflict of interest.
